# The Anti-Vivisection Movement

**Published:** 1897-03

**Authors:** 


### EDITORIAL.

The office of the Business Manager of THE JOURNAL is 311 and 312 Fitten Building.

The editors are not responsible for views expressed by contributors.

Articles for publication should reach this office not later than the 15th inst.

Address all communications, and make all remittances payable to THE ATLANTA MEDICAL AND SURGICAL JOURNAL, Atlanta, Ga.

Reprints of articles will be furnished, when desired, at a small cost. Requests for the same should always be made *on the manuscript*.

### THE ANTI-VIVISECTION MOVEMENT.

The voice of the anti-vivisectionist is being heard in the land. At this writing he is especially active in Washington, where he has succeeded in having bills prohibiting vivisection in the District of Columbia favorably reported by committees in both the House and Senate. If the efforts of these misguided and over-zealous people succeed in Washington, we may expect soon to see them show their fine hands in misdirecting legislation in the States.

The high-priest of the anti-vivisectionists is the President of the New York Society for the Prevention of Cruelty to Animals, whose only argument, in a late controversy over experiments upon animals, consisted in the simple statement, "I do not believe in such experiments." They have attempted to create the impression in Washington that a systematic torturing of animals is carried on in our medical schools, to the great delight and entertainment of admiring students, and that the students themselves are either allowed or required to perform these unnecessary and harrowing cruelties. It is safe to say that no such system of instruction is practiced anywhere in this country. We understand that the measure has found a sympathetic champion in Senator Gallinger, of New Hampshire, who is a physician, and should therefore know better.

That is a false humanity which deprives mankind to spare a dog The animals that have been sacrificed in vivisection have materially helped to establish the sciences of physiology and pathology; they have contributed no small share to our knowledge as to the prevention and cure of disease, and they have conferred certain and enduring blessings upon the human family.

The fruits of vivisection have been nowhere more clearly set forth than in a late article by Sir Andrew Clark, in the London *Hospital.* He says:

"By experimental research surgeons have been enabled to arrest or cure diseases by excising portions of the stomach, by removing one of the kidneys, by amputating the larynx, and by extirpating portions of the brain and spinal cord. By experimental research Koch discovered, in the presence and action of the tubercular bacillus, the true cause of tuberculous disease; and this discovery has not only thrown a flood of light upon the natural history of consumption, but has enabled us to discover its presence when otherwise unsuspected, and to bring about a successful revolution in the treatment of surgical tuberculosis. Furthermore, there are just reasons for expecting that the continuance of this experimental research will lead to the discovery of means for the prevention, and perhaps even the cure, of pulmonary consumption, which destroys so many of the fairest and best of our race. By experimental research we have discovered the conditions for using with efficiency and safety almost all the stronger and most useful drugs, such as digitalis, chloroform, ether, chloral, nitrite of amyl, nitro-glycerine, and many others. By experiment upon animals we have discovered the nature and relations of infectious diseases; and we have learned how, in some measure, to prevent the development and to control the spread of fevers, cholera, anthrax, and septicemia. Through experiments on animals we have received the use of the electric telegraph, and all the various services which electricity now renders to the convenience and uses of man. . . . Knowledge can be advanced now only by experiment, and if experiment is to be suppressed, and if the use of knowledge acquired in this manner is to be rejected, then it is certain that the art of preventing and of curing the diseases of men and beasts will decay; that physical and mental degeneration will follow, and that science will become the contempt and pity of the world. And if physiology, pathology and therapeutics were to continue their course without the help of experiments upon animals, the errors that would be committed in the exercise of our art would bring about greater suffering and greater unhappiness than all the vivisections which have ever been performed. Lastly, if experimental research hardens the hearts of experimenters, it is only too plain that an active antagonism to it begets a disregard of accuracy, a violation of charity, and a spirit of calumny which have no parallel among ordinary men."

On this same subject Professor Huxley wrote twelve years ago:

"Unless the fanaticism of philozoic sentiment overpowers the voice of philanthropy, and the love of dogs and cats supersedes that of one's neighbor, the progress of experimental physiology and pathology will, indubitably, in course of time, place medicine and hygiene upon a rational basis. . . . At the present time science, working in the light of clear knowledge, has attacked splenic fever and has beaten it; it is attacking hydrophobia with no mean promise of success; sooner or later it will deal, in the same way, with diphtheria, typhoid and scarlet fever. To one who has seen half a street swept clear of its children, or has lost his own by these horrible pestilences, passing one's offspring through the fire to Moloch seems humanity, compared with the proposal to deprive them of half their chances of health and life because of the discomfort to dogs and cats, rabbits and frogs, which may be involved in the search for means of guarding them."

These selections from the writings of two of England's foremost scientific men in the present decade are commended to the consideration of the "philozoic sentimentalists" now performing in Washington.

